# Editorial: Interfacial engineering of carbon-based materials for efficient energy conversion

**DOI:** 10.3389/fchem.2022.1104992

**Published:** 2022-12-02

**Authors:** Ming Ge, Xiaona Li, Minmin Wang, Tao Qian, Yulin Min, Xiaolei Yuan

**Affiliations:** ^1^ School of Chemistry and Chemical Engineering, Nantong University, Nantong, Jiangsu, China; ^2^ Department of Mechanical and Materials Engineering, University of Western Ontario, London, ON, Canada; ^3^ Shanghai Key Laboratory of Materials Protection and Advanced Materials in Electric Power, Shanghai University of Electric Power, Shanghai, China

**Keywords:** interfacial engineering, carbon-based materials, nanomaterials, synthetic strategies, energy conversion

Over the past decade, carbon-based materials, such as simplex carbon materials, heteroatom-doped carbon materials, carbon-transition metal composites, have received the increasing attentions for energy conversion owning to their low cost, good electrical conductivity, and stable structure (Sun et al., 2020). Specially, the diversity in their structure and composition could significantly enhance their applications in various fields, including nanocatalysis, energy conversion, and energy storage (Hu et al., 2021). In general, catalytic reactions usually occur on the surface or interface of electrodes. The interface structure is usually formed between carbon-based materials and other components and can be theoretically used as a channel for the transportation of electrons or intermediates. Therefore, interface engineering is one of the feasible and effective strategies to enhance the performance of nanomaterials, which is the cornerstone for the practical applications (Li et al., 2021). Based on the mentioned above, this Research Topic have successfully collected two reviews and three origin research articles based on advanced synthetic techniques, novel carbon-based nanomaterials, and interfacial engineering of carbon-based materials and their derivatives for the usage in refrigerant materials, water splitting, Li-ion batteries (LIBs), as well as aqueous rechargeable zinc-ion batteries.

For fabricating novel carbon-based nanomaterials, Liang et al. synthesized holey carbon materials with ordered sub-nanometer hole defects by the oxidative cyclodehydrogenation of polyhexaphenylbenzene precursors. They found that the narrow connection between the hexabenzocoronene subunits had weak interlayer interaction energy compared to graphene, thus leading to the easy dispersion in a wide range of solvents. The experiment results successfully exhibited the novel carbon-based nanomaterials as an effective support can be applied for various inorganic nanoparticles (NPs). For example, the composites showed high catalytic activity in the reduction of nitrophenyl when polyhexabenzocoronene network supports iron NPs. Moreover, this Research Topic is also interested in the exploration of novel supports and materials. Wang et al. fabricated three Gd-based magnetic refrigerant materials by the evolution method with H_2_L and gadolinium salt in the solution of CH_3_CN/CH_3_OH. The large values of magnetic entropy have been proved to be excellent candidates as cryogenic magnetic coolants based on Schiff ligand H_2_L. In addition, such Gd-based magnetic refrigerant materials with many carbon ligands in complexes can facilitate the formation of functional carbon-based materials.

The development of interface engineering is essential to accelerate the water splitting on the carbon-based electrodes, which involves in ligands modification strategies. Wang and Wang provided a review of the recent progress of designing mononuclear catalysts based on ligands design and preparation. In this work, they provided a coherent discussion about the availability of various activity studies for structure-containable molecular complexes. Although some common strategies for preparing metal complexes were presented, they emphasized that the synthetic feasibility and complexity of metal complexes should also be considered.

In LIBs, cathode materials usually consist of active materials, carbon-based materials and binder, and active materials could determine the changing capacity and voltage of a battery. Importantly, the unique properties of carbon-based materials make them become the promising cathode modification materials. Therefore, Zhou et al. provided a feature review that systematically outlines the significant advances of carbon-based materials for cathode materials, including layered LiCoO_2_ and LiNi_x_Co_y_Al_1-x-y_O_2_, and olivine-type LiFePO_4_. Specially, they indicated that interfacial effect between cathode materials and different carbon-based nanostructures (e.g. CNT-based networks, graphene-based architectures) can promote the formation of the conductive ion/electron transfer path and increase the charge/discharge capacity, rate and cycle performance. In addition, the challenges and perspectives of carbon-based materials in cathode materials were also provided.

Rechargeable zinc-ion batteries (RZIBs) can offer high safety, low cost, and fast charge/discharge ratings for large-scale energy storage by using aqueous electrolytes. That is because that the usage of water as aqueous electrolytes could facilitate fast ion kinetics, facile processing, reduced safety concerns, and low cost. Jiang et al. reported a facile strategy to eliminate inert Zn_4_(OH)_6_SO_4_·xH_2_O for the improvement of RZIBs according to the coordination effect by using ethylenediaminetetraacetic acid-diamine (EDTA-2Na) as a coordination additive electrolyte. The stubborn insulated Zn_4_(OH)_6_SO_4_·xH_2_O that usually deposits on the anode/electrolyte interface can be eliminated timely, leading to the enhanced performance. In this system, Zn^2+^ was coordinated with the carboxyl group of four acetyl carboxyl groups and N in C–N bonds, which can accelerate the formation of a new chelating structure, thus contributing to a new interface for dissolving stubborn deposition in the electrolyte. In general, this work provides available thought and method for the development of RZIBs with carbon-based electrodes to eliminate the insoluble depositions on anodes due to the inevitable side reactions.

We would like to thank all the authors for their meaningful work and all the reviewers for their valuable contributions to this special issue. We expect that these endeavors will pave the way for further advancements in the design and fabrication of carbon-based materials and their derivatives by using interface engineering.
